# Valorization of Sour Cherry Kernels: Extraction of Polyphenols Using Natural Deep Eutectic Solvents (NADESs)

**DOI:** 10.3390/molecules29122766

**Published:** 2024-06-11

**Authors:** Danica Božović, Ivana Dimić, Nemanja Teslić, Aleksandra Mišan, Milica Pojić, Alena Stupar, Anamarija Mandić, Sanja Milošević, Zoran Zeković, Branimir Pavlić

**Affiliations:** 1 Faculty of Technology, University of Novi Sad, Blvd. cara Lazara 1, 21000 Novi Sad, Serbia; danica_bozovic@live.com (D.B.); ivana.dimic@live.com (I.D.); sanjamilosevic9898@gmail.com (S.M.); zzekovic@tf.uns.ac.rs (Z.Z.); 2 Institute of Food Technology, University of Novi Sad, Blvd. cara Lazara 1, 21000 Novi Sad, Serbia; aleksandra.misan@fins.uns.ac.rs (A.M.); milica.pojic@fins.uns.ac.rs (M.P.); alena.stupar@fins.uns.ac.rs (A.S.); anamarija.mandic@fins.uns.ac.rs (A.M.)

**Keywords:** sour cherry kernels, hydrophilic NADES, hydrophobic NADES, polyphenols, antioxidant activity

## Abstract

The objective of this research was to optimize the natural deep eutectic solvent (NADES) extraction process from sour cherry kernels (*Prunus cerasus* L.). For polyphenol isolation, conventional solid–liquid extraction was employed using different concentrations of ethanol (0, 10, 20, 30, 40, 50, 60, 70, 80, 90, and 96%), as well as the innovative NADES extraction technique. In the initial phase of the research, a screening of 10 different NADESs was conducted, while extraction was carried out under constant parameters (50 °C, 1:20 *w*/*w*, 60 min). NADES 4, composed of lactic acid and glucose in a molar ratio of 5:1, exhibited the highest efficiency in the polyphenol isolation. In the subsequent phase of the research, response surface methodology (RSM) was utilized to optimize the extraction process. Three independent variables, namely temperature, extraction time, and solid–liquid (S/L) ratio, were examined at three different levels. The extracted samples were analyzed for total phenol (TP) and antioxidant activity using the DPPH, ABTS, and FRAP assays. ANOVA and descriptive statistics (R^2^ and CV) were performed to fit the applied model. According to RSM, the optimal extraction conditions were determined as follows: temperature of 70 °C, extraction time of 161 min, and S/L ratio of 1:25 *w*/*w*.

## 1. Introduction

The myriad agricultural and industrial processes have a tremendous impact on residual biomass generation, which, if improperly disposed, is accumulated in landfills, causing environmental concerns. Developing nations are in a constant search for sustainable solutions for the utilization and valorization of these valuable resources. The waste is usually ignited, which leads to a higher greenhouse effect, low energy capacity, health risks, and compromising the welfare of the community. Even though organic matter found in agro-industrial waste has valuable potential, there are a limited number of technologies that can ensure the safe recovery and refinement of the active ingredients [1]. Studies have shown that agri-food waste can be utilized as a resource of various bioactive compounds, including polyphenols, polysaccharides, anthocyanins, fatty acids, fibers, enzymes, and proteins. These compounds, apart from their primary nutritional role, have anti-inflammatory, antimicrobial, antioxidant, and anticancer properties that can have a positive impact on human health and help against acute and chronic illnesses [2,3,4,5,6]. Food waste and by-products are demanded to be used as a starting point for obtaining high-value-added products in the food, pharmaceutical, cosmetic, energy, and chemical industries and meet sustainability requirements in parallel, which requires considerable effort. As a result, the complexity of waste minimization, utilization of by-products, and isolation of bioactives with potential nutraceutical applications has become a great obstacle in the food industry [3].

Sour cherry (*Prunus cerasus* L.), belonging to the Rosaceae family, is cultivated on a global scale for its fruits, which are essential in the food industry for juice, jams, marmalades, alcoholic drinks, and frozen and canned food production. A considerable amount of waste in the form of kernels is obtained after the processing of cherry fruits, which presents an opportunity for the extraction of polar and non-polar compounds. The kernels are rich in different bioactive compounds, such as polyunsaturated and monounsaturated fatty acids, tocochromanols, carotenoids, phytosterols, and squalene, as well as polysaccharides and phenolic compounds. According to the literature, the presence of polar bioactive compounds such as anthocyanins in cherry kernels has also been reported [7,8,9,10].

The extraction of valuable compounds from natural resources in industry has been performed by traditional methods like Soxhlet extraction, solid–liquid extraction, hydrodistillation, and maceration. However, these techniques depend on petrochemical solvents, consume high energy, risk thermal degradation, and can last for long time periods [11]. Recent research has been conducted to improve extraction methods due to the high interest in natural compounds and environmental awareness [12]. The choice of solvents has an important role in the efficacy of extraction processes in industry, influencing the yield and quality of extracts, as well as cost and environmental pollution. Solvent waste contributes significantly to industrial contamination, and there is the need to use a lot of energy in the process of purification and recovery of solvents. Instead of using petroleum-derived solvents, scientists have been turning to the alternative green solvents because of their non-toxic nature, reusability, biodegradability, and energy-efficient synthesis [13].

A potential solution of a less harmful medium for hydrolysis and extraction processes are natural deep eutectic solvents (NADESs). These solvent mixtures can be obtained from sustainable compounds of natural origin. NADESs are formed due to the redistribution of molecular charges between hydrogen bond donors (HBDs) and acceptors (HBAs), which leads to a decrease in the melting points of NADES compounds, forming a viscous medium with a fluid-like consistency [14]. NADESs demonstrate the remarkable ability to be used as solvents for dissolving and extracting different plant-based compounds. NADESs exhibit a wide range of polarity, where depending on the nature of the compounds isolated from plant material, NADES mixtures can be polar and non-polar. Hydrophilic NADES mixtures are mainly based on choline chloride and betaine as the HBA and an organic acid as an HBD. Meanwhile, hydrophobic NADESs, which have been less researched, mostly consist of menthol as the main component in the mixture in combination with fatty acids [15,16].

Due to their unique physicochemical characteristics, it is possible to make NADES compositions that will be used for a specific application. Along with their environmental advantages, such as low vapor pressures and non-flammability, NADESs represent an optimistic choice for eco-friendly extraction methods [17]. In contrast to conventional organic solvents, NADESs seem to be a beneficial choice due to their minimal to negligible toxicity, biodegradability, cost-effectiveness, and simple preparation [18].

The main objective of this research was to valorize kernels of *Prunus cerasus* L. as a raw material for the recovery of antioxidant-rich extracts using NADESs. In the first part of the research, ten different NADESs (hydrophilic and hydrophobic) were used for screening design to determine which NADES should be used to obtain extracts with the highest total phenol content (TP) and antioxidant capacity as determined by the FRAP, DPPH and ABTS assays. The second part of the research was focused on the optimization of the extraction parameters (temperature, extraction time, solid–liquid ratio, and NADES type) using response surface methodology (RSM) to achieve the highest polyphenol recovery using TP, FRAP, DPPH, and ABTS as the target responses.

## 2. Results and Discussion

### 2.1. Comparison of Different Extraction Techniques

Preliminary studies were performed with five different hydrophilic and hydrophobic NADESs, with constant NADES extraction parameters, in order to determine the most suitable solvent for further research. In addition to NADES extraction, conventional solid–liquid extraction (SLE) was also performed, in order to obtain the extract with the highest TP content and the highest antioxidant activity, as well as to compare the efficiency of conventional and modern extraction methods. SLE extraction was performed using different ethanol concentrations (0, 10, 20, 30, 40, 50, 60, 70, 80, 90, and 96% *w*/*w*), while other extraction parameters were constant.

**Figure 1:**
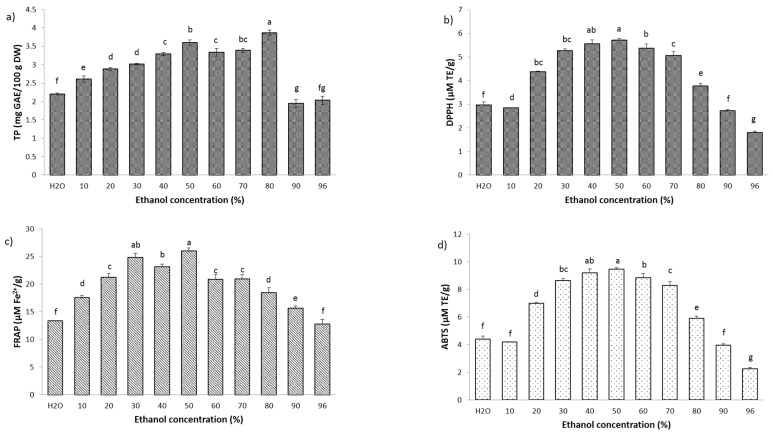
(**a**) Total phenol content, (**b**) DPPH-radical-scavenging activity, (**c**) ferric-reducing antioxidant power (FRAP) assay, and (**d**) ABTS^+^-radical-scavenging assay of cherry kernel extracts obtained by conventional solid–liquid extraction with different ethanol concentrations. Results are expressed as mean ± standard deviation (SD) and different letters represent statistically significant differences (*p* < 0.05) according to Tukey’s test.

**Figure 2:**
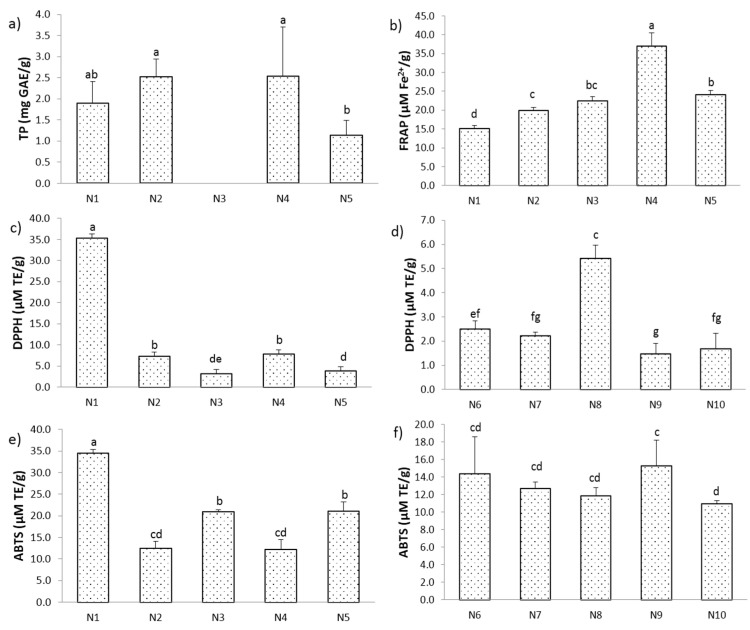
(**a**) Total phenol content, (**b**) ferric-reducing antioxidant power (FRAP) assay, (**c**,**d**) DPPH-radical-scavenging activity, and (**e**,**f**) ABTS^+^-radical scavenging-assay of cherry kernel extracts utilizing NADESs. Results are expressed as mean ± standard deviation (SD) and different letters represent statistically significant differences (*p* < 0.05) according to Tukey’s test.

The content of total phenols in the SLE extract ranged from 1.94 to 3.87 mg GAE/g DW (Figure 1a). With the increase in ethanol concentration up to a certain percentage, the content of total phenols also increased. The highest TP was obtained using 80% EtOH and was 3.87 mg GAE/g DW, while the lowest values were shown by 90% and 96% EtOH (1.94 and 2.03 mg GAE/g DW, respectively). High concentrations of ethanol may lead to dehydration and structural breakdown of plant cells, as well as the denaturation of cell wall proteins, which hinders the diffusion of polyphenols from the plant material into the extraction liquid [19]. In the case of the NADES extraction, TP was only determined for extracts obtained using hydrophilic NADES, as mixtures of lipophilic NADES were not suitable for isolating this type of compound. The total phenols for the NADES extracts ranged from 1.13 to 2.54 mg GAE/g DW. The NADES based on lactic acid and glucose (N4) in a molar ratio of 5:1 proved to be the best solvent for obtaining the highest TP values (Figure 2a). A comparative study in terms of TP was also investigated by Zhang et al. [20], where they performed extractions with seven different NADESs based on choline chloride and an extraction using 1% citric acid, water, and 70% ethanol. The results showed that the NADES composed of choline chloride:butane-1,4-diol in a molar ratio of 1:2 showed the best efficiency when isolating polyphenols from blueberry pomace. NADESs in combination with ultrasound-assisted extraction (UAE) resulted in a 1.37-fold higher yield of polyphenols than 70% ethanol as a solvent.

When it comes to antioxidant activity, the situation was different in both cases. The antioxidant activity of cherry kernel extract was determined using three in vitro assays: DPPH, ABTS, and FRAP tests. The DPPH-radical-scavenging capacity of the obtained SLE extracts ranged from 1.80 to 5.71 µM TE/g (Figure 1b). The extract obtained using 50% EtOH showed the strongest antioxidant value (*p* ≤ 0.05), while the extract obtained using 96% EtOH showed the lowest value. In the NADES extraction, the DPPH-radical-scavenging ability was best demonstrated by NADES 1, a mixture of choline chloride:malonic acid in a molar ratio 1:1 (35.26 µM TE/g), which was seven times stronger compared to the SLE extraction and NADES 4 (7.89 µM TE/g) in polar solvents. Meanwhile, NADES 8, which was composed of menthol:lactic acid in a molar ratio of 1:2, proved to be the best among the non-polar solvents in terms of antioxidant activity (5.42 µM TE/g) (Figure 2c,d).

In similar research, NADESs based on menthol:lactic acid in a molar ratio of 1:2 showed the greatest ability to remove DPPH radicals (approximately 40 mmol TE/g sample), which correlates with the results of this research [21].

The ability to remove ABTS^+^ radicals in the case of NADES extraction using polar solvents ranged from 12.24 to 34.55 µM TE/g (Figure 2e), while the antioxidant activity of the extracts obtained by non-polar NADESs varied in the range of 10.96–15.28 µM TE/g (Figure 2f). The best antioxidant activity was expressed by polar NADES 1 (34.55 µM TE/g), while the lowest antioxidant activity was shown by non-polar NADES 10 (menthol:lauric acid = 3:1). In comparison with the NADES extraction, the antioxidant activity of the SLE extraction was significantly lower. The ABTS assay was in the range of 9.45–12.24 µM TE/g (Figure 1d). The best ABTS^+^-radical-scavenging ability was shown by the extract isolated using 50% EtOH, while the weakest was shown by 96% EtOH. The same concentration of ethanol showed the highest removal power of both DPPH and ABTS^+^ radicals.

The Fe^2+^ ion reduction capacity of extracts obtained by SLE extraction ranged from 12.77 to 25.98 µM Fe^2+^/g (Figure 1c). The extract obtained with 50% EtOH showed the strongest reducing capacity (25.98 µM Fe^2+^/g), while the extract obtained with 96% EtOH showed the lowest reducing power, as in the case of the DPPH and ABTS tests. On the other hand, the NADES extracts showed a higher reducing power. The strongest reducing capacity was shown by NADES 4 and the value was 37.05 Fe^2+^/g, while the value of the weakest extract was 15.13 Fe^2+^/g, obtained using NADES 1 (Figure 2b). In terms of total phenols and antioxidant tests, the NADES extraction was found to be significantly better compared to the conventional SLE extraction.

Similar research was performed by Dulyanska et al. [22], in which the content of total phenols of cherry seed extract using different ethanol concentrations at different temperatures was determined. The best results were obtained using water:ethanol in a ratio of 60:40% at a temperature of 70 °C. Compared to this work, the TP content was slightly higher at the same EtOH concentration. Afonso et al. [23] investigated different cherry varieties. Extraction was carried out with 70% methanol using cherry kernels and stems. The TP was significantly higher in the stems than in the kernels. The highest TP in the kernels was in the Early Bigi variety, 2.76 mg GAE/g. In the case of antioxidant capacity, the same variety showed the best results. In terms of TP content and the DPPH and FRAP assays, the values were lower compared to the values in this paper, which can be explained by the use of a different solvent for the extraction. Zannou and Koca [24] reported the higher efficacy of the NADES extraction of anthocyanins from blackberry, conducting a comparison with conventional extraction methods using water, ethanol, and methanol. Regarding TP content, the best results were obtained with NADESs based on choline chloride:glucose in a molar ratio of 1:2 (9.35 mg GAE/g), while extractions with water, methanol, and ethanol yielded significantly lower results (1.87, 2.86, and 2.35 mg GAE/g, respectively), as observed in this study. Concerning antioxidative properties, DPPH and FRAP assays were performed. In the case of DPPH, the highest antioxidative activity was demonstrated by NADESs (choline chloride:citric acid 1:2), reaching 68.77 mmol TE/g. The strongest reducing capacity according to the FRAP assay was shown by the tartaric acid:xylitol NADES with a molar ratio of 1:2 (83.08 mmol ISE/g). In both cases, using methanol as a solvent among the conventional solvents, the obtained extracts exhibited the best antioxidative activity, but was significantly lower than the antioxidant activity of the NADES extracts.

### 2.2. Total Phenol Content and Antioxidant Activity

In the first step, 10 different NADES were used for extraction. NADES 4, composed of lactic acid and glucose in a molar ratio of 5:1, proved to be the optimal solvent for the isolation of polyphenols and antioxidants (Table 1). The experiments were conducted using central composed design (CCD) with three input parameters (temperature, time, and solid/liquid ratio) at three levels, evaluating four responses, which included TP and three antioxidant assays: DPPH, FRAP, and ABTS. A total of 19 runs were performed.

Observing the various conditions, the TP ranged from 3.42 to 10.27 mg GAE/g DW. The highest TP was achieved at the highest levels of the input parameters (70 °C, 180 min, and 1:30 S/L ratio), while the lowest TP value was recorded in run 2, where the temperature was 50 °C, extraction time was 60 min, and S/L ratio was 1:30. It is noticeable that the maximum temperature had a positive impact on the TP, as the highest TP values were obtained at the highest temperature levels (runs 8 and 1). Additionally, the extraction time positively influenced the TP, with the highest total phenol content obtained at the maximum time of 180 min. Based on the literature review, no studies have been found regarding the optimization of cherry kernels using NADES extraction. However, there are some studies on polyphenol extraction from various parts of berries using different extraction techniques in combination with NADESs.

The optimization of extraction by the combination of UAE and NADESs from blueberry leaves was reported by Santos-Martín et al. [25]. In this study, various NADESs were examined to identify the optimal one for the extraction of TP. After the Box–Behnken optimization, two NADESs with the highest total phenol yields were selected. The TP was 142 and 195.5 mg GAE/g for the NADES based on lactic acid:sodium acetate:water (3:1:2) and the NADES based on choline chloride:oxalic acid (1:1), respectively. According to the research carried out by Milić et al. [26], UAE was performed on vacuum-dried sour cherries using different input parameters, such as temperature, ethanol concentration, and liquid/solid ratio. The highest TP content (1.96 g GAE/100 g) was obtained at a temperature of 80 °C, with 40% ethanol, and a liquid–solid ratio of 30 mL/g. Likewise, in the case of the antioxidant tests (DPPH, FRAP, and ABTS), with the same independent variables, the highest results were obtained (69.69 µM TE/g, 51.33 µM Fe^2+^/g, and 123.45 µM TE/g, respectively).

Regarding the antioxidant tests, in this work the DPPH values ranged from 6.65 to 12.46 µM TE/g. The highest DPPH-scavenging capacity was observed in experimental run 6. The conditions yielding the highest capacity for removing free DPPH radicals were a temperature of 70 °C, extraction time of 180 min, and solid–liquid ratio of 1:10 *w*/*w*. The lowest capacity for removing DPPH radicals was observed with the lowest input parameters (50 °C, 120 min, and 1:10 S/L ratio). The maximum temperature and extraction time showed a positive influence on the ability to remove DPPH radicals. The highest Fe^2+^ removal capacity was also observed in run 6, amounting to 80.10 µM Fe^2+^/g, while the lowest results were observed in run 13, at a temperature of 60 °C, extraction time of 120 min, and solid–liquid ratio of 1:10, yielding 40.03 µM Fe^2+^/g.

In the case of the ABTS assay, the results differed significantly from the other tests. The highest ABTS-radical-scavenging capacity was achieved under the following conditions: temperature of 70 °C, extraction time of 180 min, and a 1:10 S/L ratio. The highest temperature had a significant impact on the antioxidant activity as well. The lowest antioxidant activity in the ABTS test was demonstrated in run 11, with minimal input parameters (50 °C temperature, 60 min extraction time, and 1:10 S/L ratio).

In another study by Vo et al. [27], various NADESs in combination with UAE from mulberry fruit were investigated. In terms of TP values, a NADES based on lactic acid:choline chloride in a molar ratio of 2:1 proved to be the most efficient during extraction. By applying this NADES, different factors were examined such as liquid–solid ratio, water content, time of extraction, and temperature. The highest TP value (37.7 mg GAE/g, dry basis) was obtained at an extraction temperature of 70 °C, with the optimal extraction time of 15 min, a liquid–solid ratio of 60 mL/g, and 40% water content. The strongest antioxidant activity according to the DPPH and ABTS tests was achieved under the following extraction conditions: liquid–solid ratio of 70 mL/g, 40% water content, temperature of 70 °C, and extraction time of 20 min (2.36 mM TE/g db and 207 µM TE/g db, respectively).

It could be argued that polyphenols are the major bioactive compounds responsible for in vitro antioxidant activity. This was already confirmed by our previous study focused on the application of ultrasound-assisted extraction to the recovery of polyphenols from dried sour cherries [26]. However, Ianni et al. [28] suggested that improved antioxidant activity could come as the result of a synergism between multiple compounds, other than the phenols in the mixture, capable of reacting in the free-radical-scavenging mechanism. It was already confirmed that the lipophilic fraction of cherry seeds could express significant radical-scavenging capacity towards DPPH and ABTS^+^ radicals [29]. Therefore, it is suggested that lipophilics, such as tocopherols, might contribute to the improvement of the antioxidant action of cherry seed extracts obtained by NADES extraction.

Table S1 represents the correlation between TP and antioxidant activity expressed by Pearson’s correlation coefficient (*r*). A good correlation can be observed between FRAP and TP and DPPH (*p* < 0.05). The highest DPPH value was obtained under the moderate values of all input parameters, compared to the optimal conditions required for achieving the maximum TP content. A weak correlation between these two variables, DPPH and TP, (*p* > 0.05) was proven by Pearson’s coefficient. On the other hand, the best correlation was between ABTS and TP, as well as between ABTS and FRAP, indicating a strong relationship between these factors (Table S1).

It should be highlighted that NADES extraction could provide the co-extraction of micro-, macro-, and toxic elements. In the paper of Yang et al. [30], 28 different NADESs were used for the recovery of Pb, Cd, Cr, As, and Cu from *Porphyra haitanensis*. The mixture of choline chloride and organic acids showed the best ability to recovery Pb. Furthermore, in the work of Huang et al. [31], fifteen different NADESs expressed the ability to recover 51–96% Cd from rice flour. On the other hand, it was investigated whether NADESs have the ability to co-extract trace elements from plants besides bioactive molecules. NADESs based on sucrose:lactic acid (3:1), sorbitol:lactic acid (3:1), and choline chloride:lactic acid (1:3) possessed little ability (<6%) to recovery elements, except Li, from roots of *Glycyrrhiza glabra* L. [32]. In the same study, according to an ANOVA test, the type of hydrogen bond donor expressed a significant impact on element extraction. The authors concluded that the chosen NADES were non-toxic and safe for further utilization. The detailed mechanism of binding NADES to heavy metals was explained in the paper by Lai et al. [33]. In addition to the proven fact that lactic acid has the ability to bind metals, it is necessary to emphasize the need to establish appropriate conditions, such as the solid–liquid ratio, contact time, and concentration. Based on this, it is not entirely certain that the binding of heavy metals occurs in all systems with the application of lactic acid a as hydrogen bond donor. Therefore, the investigation of the co-extraction of micro-, macro-, and toxic elements from cherry seed samples could be thoroughly investigated in our following studies.

### 2.3. Confirmation of Adequacy of Fit and Influence Analysis

To evaluate the adequacy and significance of the applied models, ANOVA (F-test) and descriptive statistics (R^2^ and CV) were used and are presented in Table 2. The coefficient of determination (R^2^) was utilized as a measure of the model’s goodness of fit. Very high R^2^ values were observed for TP and DPPH, standing at 0.9317 and 0.9208, respectively, while lower values were recorded in the case of FRAP (0.8398) and ABTS (0.8471). Generally, significant values of R^2^ indicate good agreement between the predicted model and the experimental results. In order to determine the variability of the selected responses, the coefficient of variation (CV) was used. The low CV values indicated lower variability relative to the mean value in the case of the DPPH and FRAP tests (6.33% and 9.38%, respectively), which demonstrated a good fit with adequate modeling, whereas the higher CV values obtained in the case of TP (11.69%) and ABTS (13.59%) indicated the lower precision and reliability of the experimental data.

The lack of fit test in the ANOVA suggested that the model adequately fit the experimental data, where the *p*-value (*p* > 0.05) indicated non-significant values for all response variables. The lack of fit was significant only in the case of the FRAP assay (*p* < 0.05), which did not show complete agreement between the model and the experimental data.

The application of RSM in the case of cherry kernels has not been previously investigated. However, there are similar studies that have confirmed the application of RSM for the optimization of red grape skin using NADESs with UAE [34], grape seed using UAE [35], black carrot utilizing the conventional extraction method [36], and polyphenols from grapevine canes using NADESs with UAE [37].

Based on the significance of the interaction and the linear and quadratic terms of TP, DPPH, FRAP, and ABTS, the reduced predictive model equations are presented in Table 3.

### 2.4. Influence of NADES Extraction Parameters

**Figure 3:**
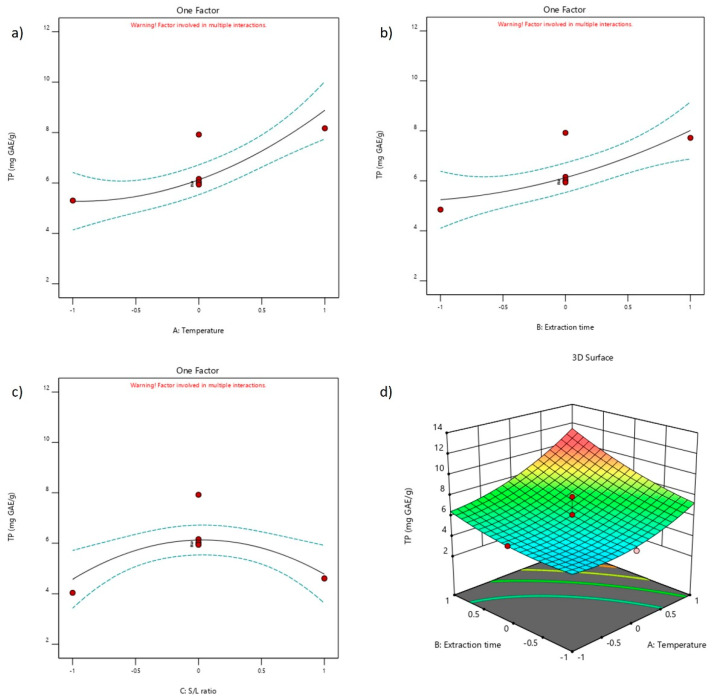
The effect of NADES extraction parameters on total phenol content (TP): (**a**) temperature, (**b**) extraction time, (**c**) S/L ratio and (**d**) temperature vs. extraction time.

**Figure 4:**
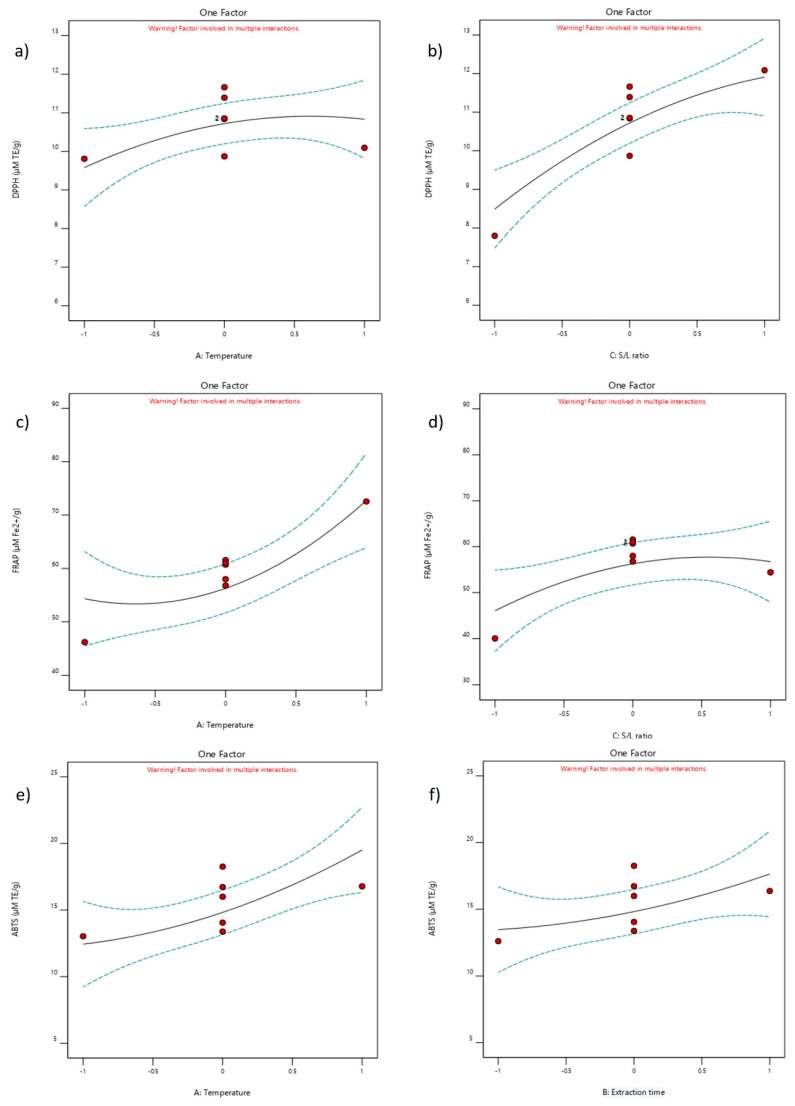
The effect of NADES extraction parameters (temperature, extraction time, and S/L ratio) on DPPH, FRAP, and ABTS assays: Influence of (**a**) temperature and (**b**) S/L ration on DPPH. Influence of (**c**) temperature and (**d**) S/L ration on FRAP. Influence of (**e**) temperature and (**f**) extraction time on ABTS.

The effect of the linear term, quadratic term, and interaction between NADES extraction parameters on TP (Figure 3) and antioxidant activity (DPPH, FRAP, and ABTS assays) (Figure 4) was observed. The TP values significantly depended on the influence of different extraction parameters compared to the other antioxidant assays. For example, positive linear terms of temperature and extraction time were observed for the TP values (Figure 3a,b). Moreover, in the 3D surface plot (Figure 3d), the interaction between temperature and extraction time leads to the conclusion that by increasing both parameters to their highest values, the maximal values for TP content can be reached. The quadratic term was dominant for the S/L ratio, in which the maximum value for the TP content was measured at the middle value for the S/L ratio (1:20 *w*/*w*), indicating that the maximal value for temperature and extraction time (70 °C and 180 min) and the middle value for the S/L ratio (1:20 *w*/*w*) should be considered the optimal set of conditions.

In three different antioxidant assays, neither the quadratic term nor the interaction between parameters was detected. Instead, only the linear term of the various parameters exhibited a significant influence on the responses. In the DPPH assay, the linear term of temperature was not dominant because the values for the responses did not rise significantly when changing the value of temperature from −1 to 1. However, the effect of the S/L ratio was more prominent than the linear term of temperature, and the large increase in the response values was measured at the highest value of the S/L ratio (1:30 *w*/*w*). The same trend of linear term effects was observed in the FRAP assay, but the temperature was the more dominant extraction parameter in this case compared to the S/L ratio. The highest value for the FRAP assay was obtained at the highest temperature. For both mentioned assays, the coded value of 1 for the linear term of temperature and the S/L ratio can be considered optimal (70 °C and 1:30 *w*/*w*). Furthermore, the 0 value for the extraction time (120 min) is suggested due to the fact that the longest extraction time was not necessary for obtaining the highest antioxidant values, which is also an economically oriented approach. The effects of the linear term of temperature and extraction time were dominant in terms of antioxidant activity against ABTS^+^ radicals. Only in this assay did the longest extraction time positively affect the ABTS value, which should be considered optimal. Other less significant extraction parameter effects on four different responses are shown in the Supplementary Material (Figures S1–S4).

Contrary to the influence of the S/L ratio on the TP content in sour cherry kernel NADES extracts, the different S/L ratio effects on the antioxidant activity could be explained by the fact that a higher S/L ratio could enable a stronger driving force and better diffusivity and dissolution based on the higher concentration gradient between the NADES and the raw material.

Moreover, the antioxidant activity of sour cherry UAE extracts was measured in the work of Milić et al. [26]. Using RSM and face-centered CCD, 16 runs were performed with temperature (40, 60, and 80 °C), ethanol concentration (40–80%, *w*/*w*), and L/S ratio (10, 20, and 30 mL/g) as independent variables. The effect of the quadratic term of all three extraction parameters did not have a significant influence. On the other hand, the linear term of temperature and the S/L ratio had a positive impact, especially at the highest values (80 °C and 30 mL/g), on the DPPH and FRAP assays. Furthermore, Milić et al. [26] observed that the extraction time had a significant influence on antioxidant activity against ABTS^+^ radicals, and the positive effect of temperature was the most dominant parameter in all three assays.

In general, for all four responses, the highest temperature could be considered the optimal value, and the same temperature expressed the highest TP and antioxidant activity in sour cherry kernel NADES extracts. Choosing a higher temperature as the optimal extraction parameter enables better mass transfer, changes the viscosity of the solvent by weakening the bonds in the NADES mixture, and facilitates the diffusion of the solvent through the sample [38].

A similar phenomenon in the case of temperature was observed in NADES extracts of strawberry tree fruit [38]. Using RSM in combination with CCD, the optimization of NADES extraction in terms of phenol content and antioxidant activity with two different solvents was performed. Temperature, extraction time, and S/L ratio were the independent variables. In both solvents (N6—betaine:glycerine:water (1:2:1) and N9—choline chloride:glycerine (1:2)), the highest temperature positively affected the FRAP and ABTS values. The linear term of the S/L ratio (1:30 *w*/*w*) was the most dominant parameter for TP content in the N6 NADES extract, and extraction time (180 min) in the N9 NADES extract. Depending on the solvent, different optimal conditions were chosen; for example, in the N6 NADES extract, the highest value for the S/L ratio enabled the highest values for all responses, as in the case of the DPPH and FRAP assays in our study. Contrary, for the N9 NADES extract, the middle value (1:20 m/m) was chosen as optimal as suggested by the TP content in sour cherry kernel NADES extracts. Similarly, for the extraction time, the middle value (150 min) for all responses was optimal for the N6 NADES extract, as for the DPPH and FRAP assays in our research. However, the highest value (180 min) for all responses in the N9 NADES extract was optimal, as in the case of TP content and the ABTS assay in sour cherry kernel NADES extracts. Teslić et al. [14] optimized the NADES extraction from defatted raspberry seeds in order to obtain the highest values for total phenols, ellagic acid, and antioxidant activity. The most dominant parameter in terms of TP content was the NADES/plant ratio (N/P), and the highest value was measured at 35 g/g instead of 15 g/g. The impact of the different values for temperature and extraction time was also investigated, and a higher temperature had a positive impact on the TP content, which is in correlation with the results in this paper. Moreover, the highest ability to neutralize DPPH radicals was observed at the lowest N/P ratio and at the highest temperature. The pattern in terms of the S/L ratio for TP and antioxidant activity in the sour cherry kernel NADES extracts was different.

Some authors optimized the process in which NADESs were used in combination with different novel extraction techniques. For example, UAE was optimized for the recovery of phenolic compounds from blueberry leaves, in which NADESs were used as a green solvent [25]. The temperature, extraction time, and L/S ratio (*v*/*w*) were the independent variables in RSM combined with the Box–Behnken design (BBD). Two NADES mixtures (lactate, sodium acetate, and water (3:1:2); choline chloride and oxalic acid (1:1)) were chosen for further process optimization. The linear term of temperature was the dominant parameter influencing TP content and antioxidant activity. Moreover, the linear term of extraction time highly affected the antioxidant activity, and the longest time was chosen as optimal. An optimal S/L ratio depends on the applied NADES mixture; for example, for the NADES based on lactic acid it was 15 (*v*/*w*), and for the NADES based on choline it was 75 (*v*/*w*). This phenomenon was correlated with the different viscosities of the NADES mixtures. Optimized microwave-assisted extraction (MAE) was used in combination with NADESs to isolate bioactive compounds with antioxidant activity from date seed powder [39]. TP content, FRAP, and DPPH assays were chosen as the responses, and temperature (20, 55, and 80 °C), irradiation time (5, 12.5, and 20 min), and percentage of NADES content (25, 55, and 85%) were the independent variables. Two different NADESs were chosen for MAE optimization and in both cases, temperature had the highest influence on the extraction process. The highest value for temperature (80 °C) was chosen as optimal in both solvents (choline chloride:lactic acid and choline chloride:xylose). The increase in TP content was achieved by increasing the temperature due to better penetration of the NADESs. Furthermore, the antioxidant activity, as expressed by the DPPH and FRAP assays, was highly dependent on the linear term of temperature and increased with increasing temperature. In order to isolate the phenolic compounds and flavonoids with antioxidant activity from black mulberry fruit, optimized UAE was performed in combination with NADESs [27]. Different values for the extraction time, temperature, water content in NADES, and L/S ratio were applied using RSM with the Box–Behnken design (BBD). The content of phenols and flavonoids increased with the increasing L/S ratio, from 10 mL/g to 60 mL/g, and decreased from 60 to 80 mL/g, and the same trend was observed for the ABTS and DPPH tests. The highest value for the temperature (70 °C) and shortened extraction time (15 min) increased the values for all responses due to degradation of bioactive compounds at extended extraction times.

### 2.5. Optimization with Multi-Response Surface Methodology

In order to optimize the NADES extraction process, RSM was employed. This statistical technique was applied to optimize process parameters such as the temperature, extraction time, and S/L ratio to obtain the highest amount of total phenols, as well as to achieve the highest antioxidant activity (DPPH, ABTS, and FRAP). The optimal conditions that resulted in the highest TP content and the highest antioxidant activity were a temperature of 80 °C, an extraction time of 121 min, and an S/L ratio of 1:25 (Table 4). The maximum predicted value according to the given model for total phenols was 10.27 mg GAE/g DW. In the case of the antioxidant assays, the values for DPPH, FRAP, and ABTS were 11.50 µM TE/g, 70.48 µM Fe^2+^/g, and 20.57 µM TE/g, respectively.

In one study, Zhang et al. [20] used RSM to optimize and validate the UAE of blueberry pomace, using DES as a solvent. The optimal extraction conditions were a water content of 29%, an L/S ratio of 36 (*v*/*w*), and an extraction temperature of 63 °C. Under these conditions, the content of TP was 41.56 ± 0.17 mg GA/g. Teslić et al. [14] also investigated the optimization and validation of NADES extraction from raspberry seeds using the Box–Behnken design (BBD) with RSM. They established two optimal conditions, the first being a temperature of 85 °C, a time of 100.45 min, and an N/P ratio of 35 g/g. Under these optimal conditions, the predicted values for TP and DPPH-radical-scavenging activity were 45.28 mg GAE/g DW and 772.52 µM TE/g DW, respectively. The experimental results for these responses were 44.77 ± 1.76 mg GAE/g DW for TP and 707.25 ± 2.87 µM TE/g DW for DPPH. The second optimal conditions were 85 °C, a time of 147 min, and an N/P ratio of 15.76 g/g. The predicted and experimental values for TP were 39.69 mg GAE/g DW and 40.16 ± 1.90 mg GAE/g DW, respectively, and for antioxidant activity, they were 1125.64 µM TE/g DW for predicted and 995.18 ± 32.08 µM TE/g DW for experimental. Regarding TP, the values were quite similar, while for the DPPH test, there were certain deviations. However, it can be concluded that the selected model was adequate for this research and that the chosen NADES proved to be effective at isolating phenols from raspberry seeds.

The optimization of NADES extraction was also reported by Milošević et al. [38] from strawberry tree fruit. Two NADESs showed good performance in terms of TP and the DPPH, ABTS, and FRAP assays. Under the specified optimal conditions, for NADES 6 (betaine:glycerine:water in a molar ratio of 1:2:1), the predicted values were 26.47 mM GAE/g (TP), 45.05 mM TE/g (DPPH), 16.00 mM Fe^2+^/g (FRAP), and 55.61 mM TE/g (ABTS), while the observed values for TP, DPPH, FRAP, and ABTS were 25.71 mM GAE/g, 40.80 mM TE/g, 15.21 mM Fe^2+^/g, and 36.54 mM TE/g, respectively. There was a noticeable deviation only in the case of the ABTS assay. Regarding NADES 9 (choline chloride:glycerine; 1:2), under the optimal conditions of 56 °C, 180 min of extraction time, and a 1:20 *w*/*w* S/L ratio, the established model proved to be efficient, and the validation of the results was carried out, showing no significant deviation in any of the responses.

In another study conducted by Vo et al. [27], NADESs in combination with UAE were investigated. Under the defined optimal conditions, the predicted value for TP was 36.6 mg GAE/g db. Meanwhile, in the case of the ABTS and DPPH assays, the values were 2.2 mM TE/g db and 187 µM TE/g, respectively. The experimental results for TP, ABTS, and DPPH were 38.6 mg GAE/g db, 2.37 mM TE/g db, and 176 µM TE/g, respectively. According to these results, there were minor differences between the predicted and experimental values, with small prediction errors.

The NADES mixtures selected in this paper are based on the literature data [40,41]. The confirmation of the NADES system for the optimal solvent used in this work (NADES 4) was performed elsewhere. On the basis of NMR analysis performed by Pisano et al. [42], it was proved that the formation of hydrogen bonds occurs in NADES 4, representing a mixture of lactic acid and glucose (5:1), which was chosen as the optimal solvent in the present work.

## 3. Materials and Methods

### 3.1. Plant Materials and Sample Preparation

The kernel, a by-product of cherry, obtained from the oil mill PAN-UNION d.o.o (Novi Sad, Serbia), was used as plant material. Primarily, the plant material was ground in a hammer mill (ABC Engineering, Pančevo, Serbia). The ground plant material was passed through the vibro-sieve set (CISA Cedaceria Industrial, Barcelona, Spain) to determinate mean particle size. The mean particle size of cherry kernels used in this research was 741 µm.

### 3.2. Chemicals

Citric acid (>99.5%) was purchased from Carl Roth, Karlsrue, Germany. From Sigma Aldrich, Sent Luis, Misuri, United States, the following reagents were supplied: glucose, sucrose, choline chloride, glycerol, 1,2-propanediol, dodecanoic acid, decanoic acid, octanoic acid, menthol, and lactic acid (natural, ≥85%). Trolox, Folin–Ciocalteu reagent, and 2,2-diphenyl-1- picrylhydrazyl (DPPH) were purchased from (Sigma Aldrich, Steinheim, Germany). Anhydrous betaine (>97%) was purchased from Tokyo Chemical Industry (Tokyo, Japan), while methanol was purchased from Macron (Avantor, Gliwice, Poland). Sodium carbonate anhydrous (>99%) was supplied from Centrohem, Stara Pazova, Serbia. Ultrapure water was obtained using the Milli-K Plus system (EMD Millipore, Billerica, MA, USA). All other chemicals used in the experiment were of analytical reagent grade.

### 3.3. Solid–Liquid Extraction (SLE)

First, 5 g of plant material was extracted with 50 mL of different concentrations of ethanol (0, 10, 20, 30, 40, 50, 60, 70, 80, 90, and 96% *w*/*w*). The extraction was performed at room temperature for 24 h, with a shaking speed of 150 rpm. Immediately after extraction, the obtained extracts were filtered through a vacuum filter. The samples were stored at 4 °C until chemical analysis.

### 3.4. Preparation of NADES

All NADESs in this work were prepared from organic acids (malonic, citric, lactic, octanoic, lauric, and decanoic) as the HBD and betaine, choline chloride, and glucose as the HBA (Table 5). NADESs were prepared in different molar ratios. Water was added to the first 5 out of 10 NADESs, with a percentage content of 20%. All NADESs were prepared by heating in a water bath at 80 °C for about 10 min, while stirring with a magnetic stirrer, until a homogeneous and stable mixture was obtained.

### 3.5. NADES Extraction

For extraction by polar (hydrophilic) NADESs, 0.05 g of cherry kernel was mixed with 1 g of NADES in glass vials with lids. The extraction was performed by stirring in a heated water bath at 50 °C placed on a magnetic stirrer (LLG-uniSTIRRER 7, Meckenheim Germany), for 60 min. After extraction, 4 g of water was added to the extracts and the mixture was centrifuged at 4000× *g* rpm for 15 min (HERMLE Z 206 A, Wehingen, Germany). After centrifugation, the supernatant was decanted into a cuvette and stored in a fridge, at 4 °C until chemical analysis. In the case of extraction with non-polar (hydrophobic) NADESs, 0.1 g of plant material and 2 g of solvent were mixed in glass vials with caps. The extraction process was performed in the same way as with the polar solvents. However, after the extraction process, water was not added to the obtained extracts. Each extraction was performed in two parallel probes.

In the first step of the experiment, a screening of NADESs was carried out, where 10 different NADESs were applied to the extraction of cherry kernels under constant conditions: temperature at 50 °C, sample/solvent ratio (S/L) of 1:20 *w*/*w*, and extraction time of 60 min. Considering that NADES 4, composed of lactic acid and glucose in a 5:1 molar ratio, proved to be the most effective solvent in terms of total phenols, further investigations were carried out using response surface methodology (RSM). In the second step, the aforementioned NADES 4 was used for extraction, where three different parameters were used at three different levels: temperature (50, 60, and 70 °C), S/L ratio (1:10, 1:20, and 1:30; *w*/*w*) and extraction time (60, 120, and 180 min). After extraction, 4 g of water was added to the obtained extracts, and the mixture was centrifuged at 4000× *g* rpm for 15 min. In the third and last step, the optimization of the applied extraction parameters was performed, regarding TP and antioxidant tests (DPPH, ABTS, and FRAP). The optimal conditions for NADES extraction were determined to be 70 °C, a 1:25 S/L ratio, and 161 min of extraction time.

### 3.6. Determination of the Total Phenol Yield (TP)

The TP in the extracts obtained by NADES extraction and solid–liquid extraction were determined by the Folin–Ciocalteu spectrophotometric method, which was explained by Singleton and Rossi [43]. Gallic acid was used as a standard for the preparation of the calibration curve. Total phenols were measured at 750 nm, and all samples were performed in triplicate. Results of total phenols were expressed as mg of gallic acid equivalents (GAEs) per g of sample dry weight (DW) (mg GAE/g DW).

### 3.7. In Vitro Antioxidant Activity Assays

#### 3.7.1. DPPH-Radical-Scavenging Assay

The method of measuring a sample’s ability to scavenge 1,1-diphenyl-2-picrylhydrazyl (DPPH) free radicals was originally presented by Brand-Williams et al. [44]. A methanol solution of DPPH reagent was freshly prepared, and its absorbance was adjusted to 0.70 ± 0.02 with methanol. The DPPH reagent (2.9 mL) was mixed with the extract samples (0.1 mL) and incubated at room temperature for 60 min, protected from light. All measurements were performed in triplicate at a wavelength of 517 nm, using a VIS spectrophotometer (6300 Spectrophotometer, Jenway, UK). The results of the antioxidant activity of the extracts were expressed as μM Trolox per g of sample (μM Trolox/g).

#### 3.7.2. ABTS^+^-Radical-Scavenging Assay

The ability to neutralize ABTS^+^ radicals was determined by a spectrophotometric method [45]. The ABTS reagent solution was prepared by mixing a 7 mM aqueous solution of 2,2′-azino-bis (3-ethylbenzothiazoline-6-sulfonic acid) diammonium salt (ABTS) and 2.45 mM potassium persulfate in a ratio of 1:1 (*v*/*v*) and incubated at room temperature for 16 h, protected from light. The ABTS reagent was diluted with acetate buffer (pH = 3.6) to adjust the absorbance to 0.70 ± 0.02. The ABTS reagent (2.9 mL) was mixed with extract samples (100 μL) and incubated at room temperature for 300 min. All measurements were performed in triplicate at 734 nm, using a VIS spectrophotometer (6300 Spectrophotometer, Jenway, UK). The results of the antioxidant activity of the obtained extracts were expressed as μM Trolox per g of sample (μM Trolox/g).

#### 3.7.3. Ferric-Reducing Antioxidant Power (FRAP) Assay

The ability to reduce Fe^3+^ ions was determined by the ferric-ion-reducing antioxidant power (FRAP) assay [46]. This method was performed for extracts isolated using polar solvents, while in the case of the extracts obtained with non-polar solvents, the FRAP assay was not conducted. The main reason for this was the inability to isolate compounds capable of reducing Fe^3+^ ions with non-polar NADESs from plant material. The FRAP reagent, which is crucial for the analysis, was prepared by mixing 10 mM/L 2,4,6-tripyridyl-s-triazine (TPTZ) in 40 mM/L HCl, 20 mM/L FeCl_3_, and acetate buffer (pH 3.6), in a ratio of 1:1:10, respectively. Then, 2.9 mL of the reagent was mixed with 0.1 mL of the obtained extract and incubated in the dark at a temperature of 37 °C for 10 min. After that, the absorbance was measured at a wavelength of 593 nm. The results were expressed as µM Fe^2+^ equivalents per gram of sample (µM Fe^2+^/g).

### 3.8. Experimental Design and Statistical Analysis

Response surface methodology (RSM) is a widely used method that serves to examine the influence of parameters on the extraction process and to optimize the process. In this work, optimization was performed using RSM in combination with a central composed design (CCD). Temperature (50, 60, and 70 °C), extraction time (60, 120, and 180 min) and S/L radio (1:10, 1:20, and 1:30 *w*/*w*) were the independent variables, which were investigated by the aforementioned method for optimization. In order to normalize the parameters, each of the coded variables was forced into range from −1 to 1, so that they all affected the response more evenly and thus the units of the parameters were irrelevant. Variables were coded according to the following equation:

X = (x_i_ − x_0_)/∆x (1)

where X is the coded value, x_i_ is the corresponding actual value, x_0_ is the actual value in the center of the domain, and Δx is the increment of x_i_ corresponding to a variation of 1 unit in X.

A total of 19 experiments were carried out with 4 responses, including TP (mg GAE/g), DPPH (µM TE/g), FRAP (µM Fe^2+^/g) and ABTS (µM TE/g). The second-order polynomial model was used in the response surface analysis to estimate the effect of each extraction factor (Equation (2)):

(2) $$Y=\beta_{0}+\sum_{i=1}^{5}\beta_{i}X_{i}+\sum_{i=1}^{4}\sum_{j=i+1}^{5}\beta_{ij}X_{i}X_{j}$$

where Y represents the response, and β_0_, β_i_, β_j_, and β_ij_ are the intercept, linear, quadratic, and interactive regression coefficients of the model, respectively. X_i_ and X_j_ represent independent variables. RSM analysis was performed using Design-Expert v.11 software (Stat-Ease, Minneapolis, MN, USA). The first part of the study, involving the screening of NADESs, utilized one-way analysis of variance (ANOVA) (*p* ≤ 0.05) with the post-hoc Tukey test using Statistica 13 Software (Statsoft Inc., Tulsa, OK, USA). ANOVA with a significance level of 0.05 was used to determine the goodness of fit. The adequacy of the model was described and evaluated by the coefficient of determination (R^2^), coefficient of variance (CV), and *p*-values for the model, as well as lack of fit.

## 4. Conclusions

In this study, the cherry kernel was utilized as a plant material and as a by-product with the aim of reducing industrial waste, while simultaneously isolating bioactive compounds from the investigated part of the plant. Two types of extractions were conducted: a conventional extraction using different concentrations of ethanol (0, 10, 20, 30, 40, 50, 60, 70, 80, 90, and 96%) and an innovative NADES extraction technique. In the case of the NADES extraction, ten different NADESs were used, consisting of five polar and five non-polar solvents. In the first part of the research, the NADES screening determined which solvent was the most suitable for further research in terms of the highest amount of total polyphenols. Extractions using various solvents were performed under constant conditions, whereby NADES 4 (lactic acid and glucose in a molar ratio of 5:1) emerged as the most suitable solvent in terms of TP. In further research, RSM in combination with CCD was applied. Three independent variables (temperature, S/L ratio, and extraction time) were selected for investigation at three different levels. Experiments were performed with four responses, including TP, and three antioxidant assays (DPPH, FRAP and ABTS). According to experimental design, the optimal conditions were 70 °C, 161 min, and a 1:25 *w*/*w* S/L ratio, aimed at maximizing the examined values. In this study, the NADES extraction proved to be more efficient in isolating polyphenols and antioxidant capacity compared to the conventional extraction. NADES 4 emerged as the most effective in terms of the TP and FRAP methods, while NADES 1 (choline chloride:malonic acid = 1:1) also demonstrated efficacy in removing free DPPH and ABTS radicals. It can be concluded that cherry kernels represent a rich source of polyphenols, and their extracts are rich in antioxidants. Additionally, the NADES extraction proved to be a safe, innovative, and efficient method, yielding non-toxic extracts that could be further applied to various branches of industry.

## Figures and Tables

**Table 1:** Central composite experimental design with coded extraction parameters of NADES 4 and experimentally observed values of target responses: total phenol content and antioxidant activity (DPPH, FRAP, and ABTS).

	Input Factors			Response 1	Response 2	Response 3	Response 4
Run	A: Temperature [°C]	B: Extraction Time [min]	C:S/L Ratio [g/g]	TP [mg GAE/g DW]	DPPH [µM TE/g]	FRAP [µM Fe^2+^/g]	ABTS [µM TE/g]
1	1	0	0	8.17	10.09	72.54	16.78
2	−1	−1	1	3.42	9.80	55.94	9.22
3	0	0	0	5.94	9.87	60.97	14.05
4	1	−1	−1	5.50	8.08	63.46	14.91
5	0	0	0	6.16	11.66	61.55	13.38
6	1	−1	1	6.59	12.46	80.10	19.96
7	0	0	0	6.03	10.85	57.99	16.73
8	1	1	−1	10.09	8.38	62.34	21.10
9	0	1	0	7.72	10.44	55.38	16.37
10	0	0	0	7.92	10.84	56.78	16.00
11	−1	−1	−1	3.86	6.65	41.57	7.68
12	1	1	1	10.27	11.84	63.86	20.16
13	0	0	−1	4.04	7.80	40.03	10.31
14	0	−1	0	4.85	9.87	47.89	12.61
15	−1	0	0	5.30	9.81	46.19	13.03
16	0	0	1	4.61	12.08	54.42	13.39
17	0	0	0	5.97	11.39	60.71	18.26
18	−1	1	−1	5.20	8.26	49.97	15.17
19	−1	1	1	4.79	10.08	56.51	12.46

TP—total phenol content; DPPH—2,2-diphenyl-1-picrylhydrazyl; ABTS—2,2-azino-bis (3-ethylbenzothiazoline-6-sulphonic acid); FRAP—ferric-ion-reducing antioxidant power; GAE—gallic acid equivalent; TE—Trolox equivalent.

**Table 2:** Analysis of variance (ANOVA) and descriptive statistics (R^2^ and CV) of the fitted model.

Response	Source	SS	df	MS	F-Value	*p*-Value
TP	Model	63.00	9	7.00	13.65	0.0003
	Residual	4.62	9	0.5128		
	Lack of Fit	1.71	5	0.3412	0.4692	0.7860
	Pure Error	2.91	4	0.7273		
	Cor Total	67.61	18			
	R^2^	0.9317				
	CV [%]	11.69				
DPPH	Model	41.99	9	4.67	11.63	0.0006
	Residual	3.61	9	0.4013		
	Lack of Fit	1.72	5	0.3450	0.7312	0.6366
	Pure Error	1.89	4	0.4718		
	Cor Total	45.60	18			
	R^2^	0.9208				
	CV [%]	6.33				
FRAP	Model	1451.25	9	161.25	5.24	0.0107
	Residual	276.89	9	30.77		
	Lack of Fit	259.47	5	51.89	11.92	0.0162
	Pure Error	17.42	4	4.36		
	Cor Total	1728.14	18			
	R^2^	0.8398				
	CV [%]	9.68				
ABTS	Model	202.07	9	22.45	5.54	0.0089
	Residual	36.49	9	4.05		
	Lack of Fit	20.72	5	4.14	1.05	0.4944
	Pure Error	15.77	4	3.94		
	Cor Total	238.55	18			
	R^2^	0.8471				
	CV [%]	13.59				

SS—sum of squares; CV—coefficient of variation; MS—mean square; df—degrees of freedom; F—F-value; *p*—*p*-value.

**Table 3:** The fitted quadratic polynomial regression model equation for TP, DPPH, FRAP, and ABTS.

Response	Equation
TP	TP = 6.13 + 1.81A + 1.39B+ 0.6943AB + 0.9544A^2^ − 1.46C^2^
DPPH	DPPH = 10.72 + 0.6264A + 1.71C
FRAP	FRAP = 56.27 + 9.21A + 5.35C + 7.26A^2^
ABTS	ABTS = 14.83 + 3.54A + 2.09B

**Table 4:** Optimized NADES parameters of polyphenols and antioxidant activity by RSM approach.

Input and Output Parameters	Predicted Values
Temperature [°C]	70
Extraction time [min]	161
S/L ratio [g NADES/g]	1:25
TP [mg GAE/g DW]	10.27
DPPH [µM TE/g]	11.50
FRAP [µM Fe^2+^/g]	70.48
ABTS [µM TE/g]	20.57

**Table 5:** List of 10 NADESs prepared and used in this research.

Mark	Sample		Molar Ratio	Water Content [%]	NADES Type
	Hydrogen Bond Acceptor	Hydrogen Bond Donor			
N1	Choline chloride (ChCl)	Malonic acid (MA)	1:1	20	Hydrophilic
N2	Betaine (Bet)	Citric acid (CA)	1:3	20	Hydrophilic
N3	Betaine (Bet)	Glycerol (Gly)	1:2	20	Hydrophilic
N4	Glucose (Glu)	Lactic acid (LA)	5:1	20	Hydrophilic
N5	Choline chloride (ChCl)	1,2-Propanediol (PD)	1:1	20	Hydrophilic
N6	Octanoic acid (OA)	Lauric acid (LA)	3:1	/	Hydrophobic
N7	Decanoic acid: (DA)	Lauric acid (LA)	3:1	/	Hydrophobic
N8	Menthol (Menthol)	Lactic acid (LA)	1:2	/	Hydrophobic
N9	Menthol (Menthol)	Decanoic acid (DA)	7:2	/	Hydrophobic
N10	Menthol (Menthol)	Lauric acid (LA)	3:1	/	Hydrophobic

## Data Availability

The data presented in this study are available in article and Supplementary Materials.

## Supplementary Materials

The following supporting information can be downloaded at: https://www.mdpi.com/article/10.3390/molecules29122766/s1. Figure S1. Other less significant effect of NADES extraction parameters (temperature, extraction time and S/L ratio) on total phenolic content (TP); Figure S2. Other less significant effect of NADES extraction parameters (temperature, extraction time and S/L ratio) on DPPH assay; Figure S3. Other less significant effect of NADES extraction parameters (temperature, extraction time and S/L ratio) on FRAP assay; Figure S4. Other less significant effect of NADES extraction parameters (temperature, extraction time and S/L ratio) on ABTS assay; Table S1. Correlation matrix with Pearson’s coefficient of correlation for TP, DPPH, FRAP and ABTS.
