# Supplementary material for: Valorization of Sour Cherry Kernels: Extraction of Polyphenols Using Natural Deep Eutectic Solvents (NADESs)
Source: Molecules. 2024 Jun 11;29(12):2766. doi: 10.3390/molecules29122766 (PMC11206417; doi:10.3390/molecules29122766)
Supplement: Supplementary file 1 [file molecules-29-02766-s001.zip › molecules-3002342-supplementary.pdf]

Supplementary material

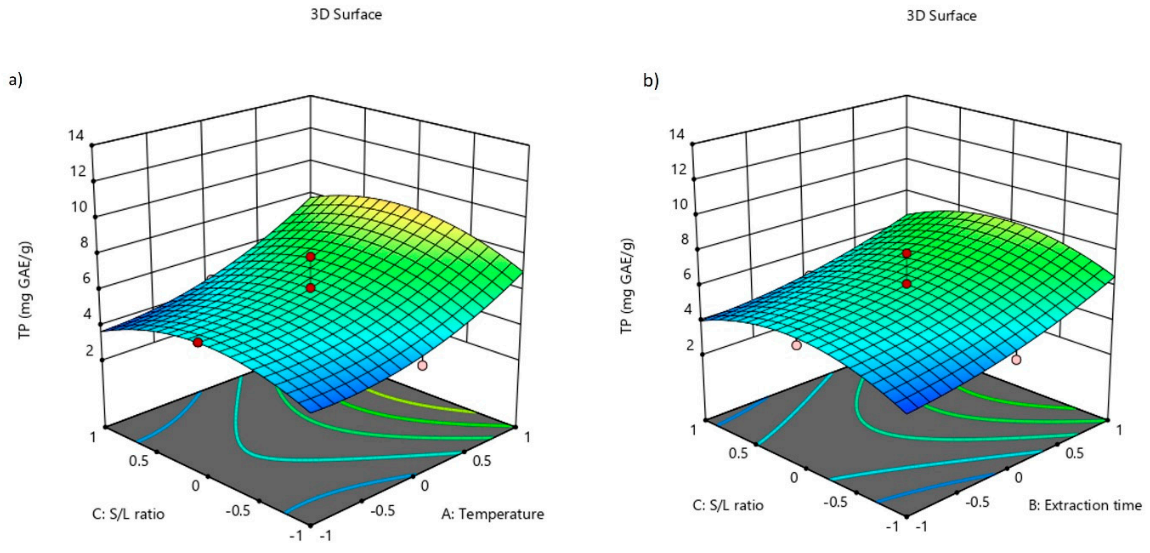

Figure S1. (a,b) Other less significant effect of NADES extraction parameters (temperature, extraction time and S/L ratio) on total phenolic content (TP)

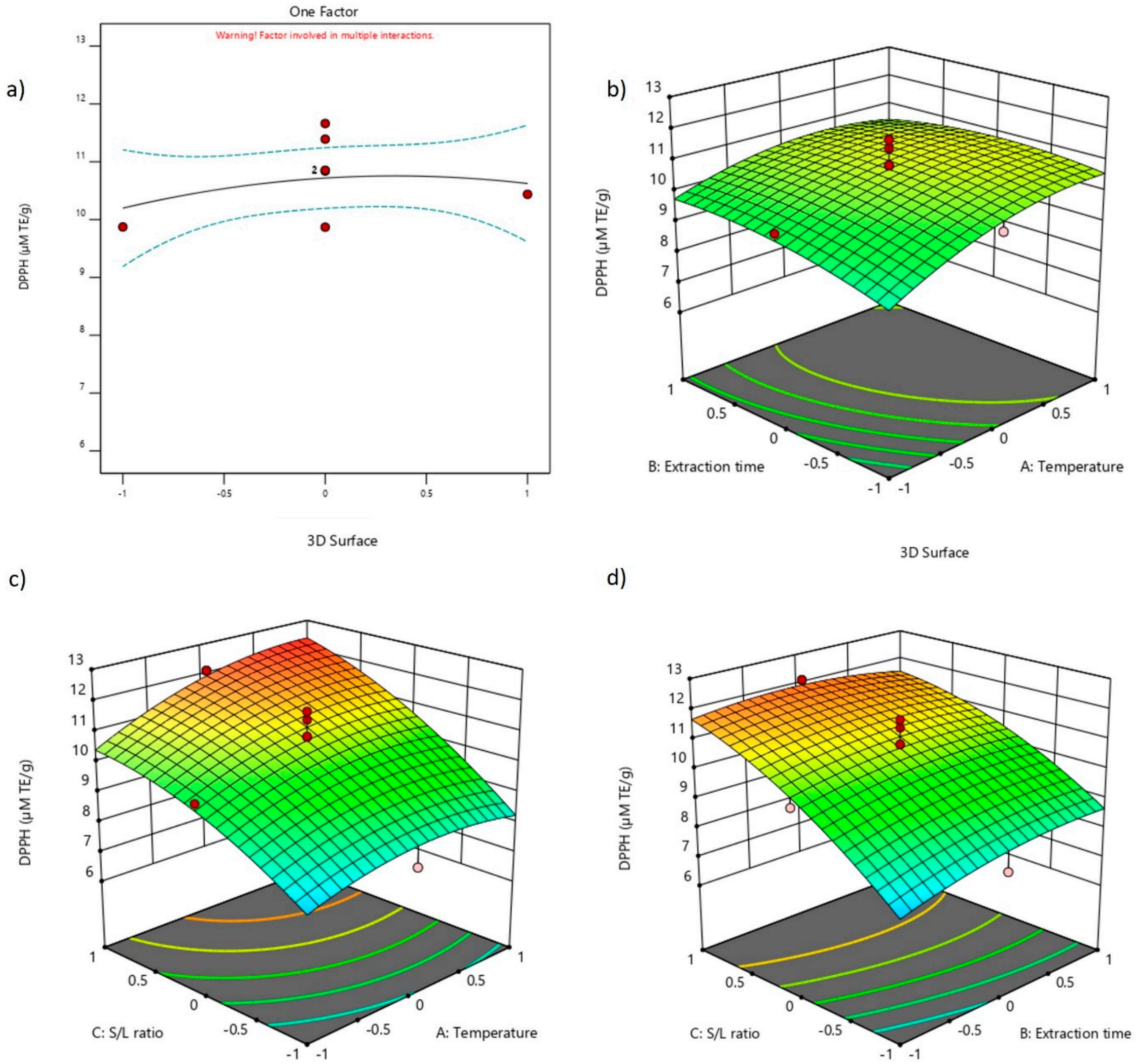

Figure S2. (a-d) Other less significant effect of NADES extraction parameters ((a) temperature, (b) extraction time (c,d) and S/L ratio) on DPPH assay

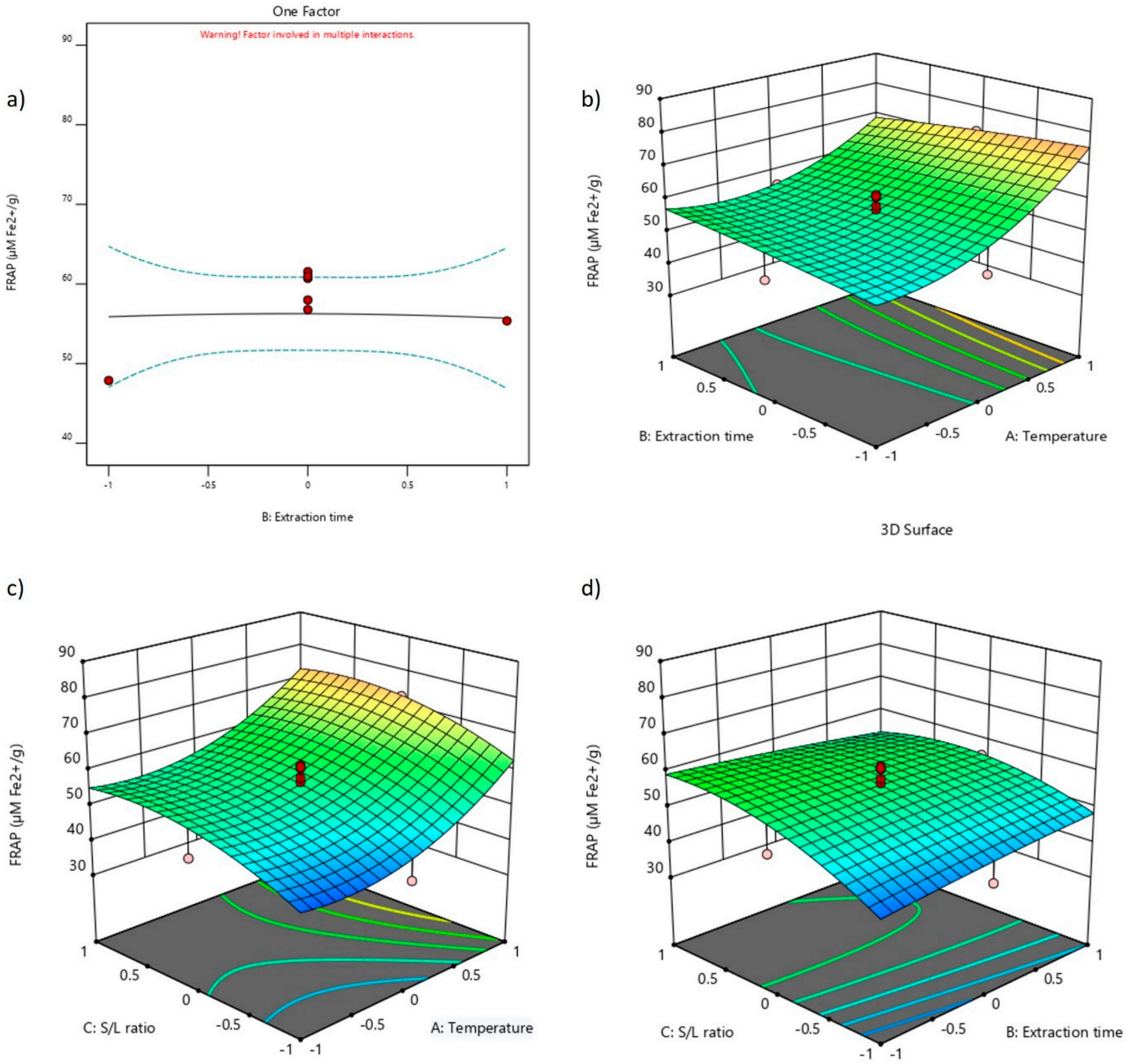

Figure S3. (a-d) Other less significant effect of NADES extraction parameters ((a) temperature, (b) extraction time and (c,d) S/L ratio) on FRAP assay

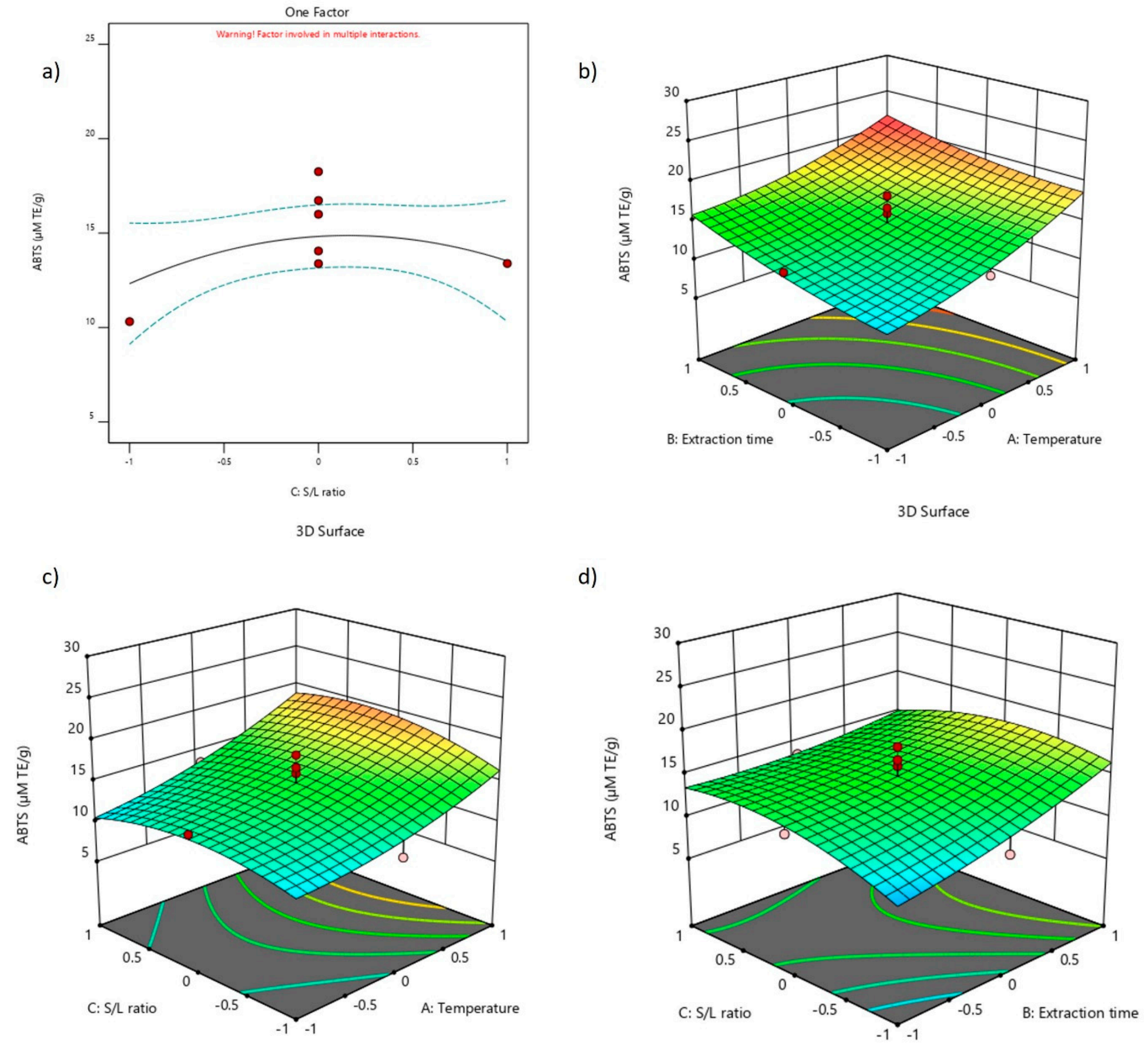

Figure S4. (a-d) Other less significant effect of NADES extraction parameters (temperature, extraction time and S/L ratio) on ABTS assay

Table S1. Correlation matrix with Pearson's coefficient of correlation for TP, DPPH, FRAP and ABTS

r	TP	DPPH	FRAP	ABTS
TP	1			
DPPH	0.308	1		
FRAP	0.552*	0.574*	1	
ABTS	0.845*	0.478*	0.711*	1

r - Pearson's correlation coefficient
$^*p < 0.05$
